# Treatment Options for Paediatric Anaplastic Large Cell Lymphoma (ALCL): Current Standard and beyond

**DOI:** 10.3390/cancers10040099

**Published:** 2018-03-30

**Authors:** Nina Prokoph, Hugo Larose, Megan S. Lim, G. A. Amos Burke, Suzanne D. Turner

**Affiliations:** 1 Division of Cellular and Molecular Pathology, Department of Pathology, University of Cambridge, Cambridge CB2 0QQ, UK; np465@cam.ac.uk (N.P.); hl457@cam.ac.uk (H.L.); 2 Department of Pathology and Laboratory Medicine, University of Pennsylvania, Philadelphia, PA 19104, USA; megan.lim@uphs.upenn.edu; 3 Department of Paediatric Haematology, Oncology and Palliative Care, Addenbrooke’s Hospital, Cambridge University Hospitals NHS Foundation Trust, Cambridge CB2 0QQ, UK; ab2359@cam.ac.uk

**Keywords:** ALCL99, alectinib, brentuximab vedotin (BV), crizotinib, nivolumab, NPM-ALK, pediatric, SGN-35, Tyrosine Kinase Inhibitor (TKI)

## Abstract

Anaplastic Lymphoma Kinase (ALK)-positive Anaplastic Large Cell Lymphoma (ALCL), remains one of the most curable cancers in the paediatric setting; multi-agent chemotherapy cures approximately 65–90% of patients. Over the last two decades, major efforts have focused on improving the survival rate by intensification of combination chemotherapy regimens and employing stem cell transplantation for chemotherapy-resistant patients. More recently, several new and ‘renewed’ agents have offered the opportunity for a change in the paradigm for the management of both chemo-sensitive and chemo-resistant forms of ALCL. The development of ALK inhibitors following the identification of the EML4-ALK fusion gene in Non-Small Cell Lung Cancer (NSCLC) has opened new possibilities for ALK-positive ALCL. The uniform expression of CD30 on the cell surface of ALCL has given the opportunity for anti-CD30 antibody therapy. The re-evaluation of vinblastine, which has shown remarkable activity as a single agent even in the face of relapsed disease, has led to the consideration of a revised approach to frontline therapy. The advent of immune therapies such as checkpoint inhibition has provided another option for the treatment of ALCL. In fact, the number of potential new agents now presents a real challenge to the clinical community that must prioritise those thought to offer the most promise for the future. In this review, we will focus on the current status of paediatric ALCL therapy, explore how new and ‘renewed’ agents are re-shaping the therapeutic landscape for ALCL, and identify the strategies being employed in the next generation of clinical trials.

## 1. Clinical Features of Paediatric Paediatric Anaplastic Large Cell Lymphoma (ALCL)

In 1982, Stein and colleagues described tumours composed of neoplastic cells of unknown origin found in what was thought to be Hodgkin’s Lymphoma (HL), expressing the CD30 antigen (Ki-1, Ber-H2) [1,2]. These tumours were initially known as Ki-1 positive Large Cell Lymphomas. Further immunohistochemical and molecular analysis of these tumours, particularly the identification of the characteristic translocation (t(2;5)(p23;q35)) and the successful cloning of the breakpoints by Steve Morris and Tom Look in 1994, revealing fusion of the nucleolar phosphoprotein gene *nucleophosmin 1* (*npm1*) with that of a newly described gene, *anaplastic lymphoma kinase* (*alk*), led to the establishment of the entity now known as ALK-positive Anaplastic Large Cell Lymphoma (ALCL) [3,4,5,6]. Over the next decade, the two provisional entities known as ALK-positive and ALK-negative ALCL were proposed and finally adopted into the WHO classification of tumours of haemopoietic and lymphoid tissues in 2017 [7].

ALCL is primarily a paediatric tumour, accounting for 15% of all paediatric Non-Hodgkin Lymphoma (NHL) with an annual incidence ranging from 1.2 per million in children under 15 years, to approximately 2 per million in young adults between 25 and 34 years [8], translating to approximately 80 new paediatric cases diagnosed in Europe each year [9]. Whilst the majority of paediatric cases are ALK-positive, about 50–60% of adult ALCL cases are ALK-negative. It is estimated that 90% of paediatric ALCL show aberrant expression of ALK fusion proteins, and of those, approximately 75% express NPM-ALK [10]. ALK-positive ALCL show improved chemo-responsiveness and patients experience superior survival compared with ALK-negative disease. Age may be a confounding factor in the poorer prognosis of ALK-negative disease [11]. However, considering only paediatric cases, overall survival (OS) rates are still higher for ALK-positive paediatric patients than for ALK-negative ones, with an event-free survival (EFS) of 65–75% for ALK-positive ALCL depending on the treatment regimen, compared to 15–46% for ALK-negative ALCL [12,13,14,15].

## 2. Frontline Treatment for Paediatric ALCL

Fortunately, paediatric ALCL patients are relatively chemo-sensitive with high response rates to diverse chemotherapy regimens, as proven by various studies; EFS and OS vary between 65% and 75%, and 70% and 90%, respectively, independent of treatment duration, drugs used, or their dosages (Table 1) [13,14,15,16,17,18].

Given that ALCL was not recognised as a distinct form of NHL until 1989, most patients prior to this time would have been treated as B or T-cell NHL. The NHL-Berlin-Frankfurt-Münster (NHL-BFM) working group enrolled paediatric patients with B or T cell NHL into three different trials: NHL-BFM83, NHL-BFM86, or NHL-BFM90 [15,19,23]. Though the trials were not primarily aimed at ALCL, a retrospective analysis revealed an 83% 9-year EFS, and an OS of 81% for CD30-positive ALCL patients [19]. NHL-BFM90 was the first trial to include a treatment arm specifically for ALCL, although presence of the ALK translocation was not used as an inclusion criteria [15]. The treatment protocol was based on the previous NHL-BFM studies (Table 2).

Patients were enrolled into one of three arms according to disease stage: arm K1 for stages I and II if completely resected (nine patients), K2 for stage II non-resected and stage III (65 patients), and K3 for stage IV (14 patients). Because CD30-positive ALCL resembled B-cell NHL closely, the first protocol trialled was that used for B-cell NHL, which used methotrexate. Thus, the arms K1 to K3 tested increasing doses of methotrexate. NHL-BFM90 led to a 5-year EFS of 100%, 73%, and 79% respectively for arms K1, K2, and K3. The treatment regimen lasted between 2 and 5 months compared to 7 or 8 months respectively for HM89 and HM91 (Table 1), which are both protocols that were tested by the French Society for Paediatric Oncology (SFOP) at that time. As a result, and because the drug doses were comparatively lower—all with comparable EFS rates—the NHL-BFM working group recommended its NHL-BFM90 protocol as standard therapy for ALCL [13,15,27,28].

Given the high risk of short-term side effects associated with methotrexate such as oral and gastrointestinal mucositis, sometimes leading to sepsis and toxic death [24], lower concentrations of methotrexate administered in shorter pulses were investigated in the subsequent NHL-BFM95 trial (Table 2). NHL-BFM95 stratified patients into low risk (stages I and II, arms R1 and R2) and high-risk patients (stages III and IV, arms R3 and R4). Patients in arms R1/R2 and R3/R4 were treated with 1 g/m^2^ and 5 g/m^2^ methotrexate infusions, respectively. In both cases, half the patients were randomized to receive the infusion over 4 h, whilst the other half were given the infusion over 24 h. The trial found that the 4-h infusion and the 1 g/m^2^ dose were not inferior but were less toxic than the 24-h infusion and 5 g/m^2^ injection [24].

The European Inter-group for Childhood Non-Hodgkin Lymphoma (EICNHL) launched the first international randomized trial for ALCL patients under 22 years of age, regardless of ALK status in 1999—the ALCL99 trial (NCT00006455) [10,29,30]. ALCL99 enrolled 352 children over 7 years in 11 European countries and Japan. The trial tested four different protocols aiming to achieve three main goals: to lower the amount of methotrexate required, to rid the protocol of intrathecal injections, and to test whether vinblastine could be a valuable addition to the protocol. Patients were randomly enrolled into arms MTX1 and MTX3, which tested the NHL-BFM90 backbone with a 24-h low-dose (1 g/m^2^) methotrexate infusion (without intrathecal injections) and intermediate-dose (3 g/m^2^) 3-h methotrexate infusion (with intrathecal injections), respectively. The trial achieved a 2-year EFS of 74.1% and a 2-year OS of 92.5%, and found that the MTX3 arm using a higher dose, but a shorter infusion time for methotrexate was overall less toxic than the MTX1 arm [10,12,13,14,15,31]. Thus, the investigators recommended using short-pulse, high-dose methotrexate without intrathecal injections for reduced toxicity and improved quality of life. This has become the chemotherapy regimen referred to hereafter as ALCL99 [10,31,32].

Besides the observed short-term toxicity, relapse following ALCL99 was comparable with previous trials (HM89, HM91, NHL-BFM83, NHL-BFM86, and NHL-BFM90) averaging at 20–40% with some children experiencing multiple events [10]. However, whilst these children tend to remain chemo-sensitive, they still suffer the long-term side effects of toxic chemotherapy [31].

### 2.1. Vinblastine: Potential New Paradigm

Two small retrospective studies conducted by the SFOP showed that vinblastine could reduce the risk of treatment failure, even for patients who had relapsed on chemotherapy [33,34]. Hence, as part of the ALCL99 protocol, vinblastine was investigated in high-risk patients (those with mediastinal, lung, liver, or spleen involvement, or biopsy-proven skin lesions) who were eligible for the sub-trial, ALCL99-VBL (Table 1 and Table 2). High-risk patients were first randomized into one of MTX1-VBL or MTX3-VBL arms, and then half were randomly selected to receive weekly Vinblastine at 6 mg/m^2^, in addition to the MTX1 or MTX3 protocol they were already in, followed by weekly vinblastine-only injections for 1 year on its own as a maintenance treatment [21]. Results showed a significant improvement over the first year of treatment with regard to EFS, but no significant difference overall with relapse being delayed rather than prevented [21]. Vinblastine was also investigated as frontline therapy in the Children’s Oncology Group (COG) trial ANHL0131 (NCT00059839), in addition to the chemotherapy backbone (APO: doxorubicin, prednisone, vincristine, methotrexate, 6-mercaptopurine). Similar to the European trial, it did not find any significant difference between 3-year OS or EFS as compared to standard chemotherapy, but did show that weekly vinblastine administration was more toxic than the ‘no vinblastine’ arm [22]. For both ANHL0131 and ALCL99-VBL, the vinblastine dose started at 6 mg/m², but had to be reduced to 4 mg/m² due to toxicity in the majority of patients (41/61).

However, mainly in the context of relapsed disease, accumulating evidence suggests that vinblastine has unusual efficacy as a single agent in ALCL when given for prolonged durations [21,33,34]. The EICNHL-ALCL-RELAPSE trial included an arm that recruited patients with late relapse (more than 12 months from initial diagnosis) and CD3-negative ALCL treated with single agent weekly vinblastine for 24 weeks. A first abstract in the British Journal of Haematology reports that vinblastine achieved both high survival rates and length of remission, sometimes for the entire 24 months duration of the treatment [35].

This experience with single agent vinblastine in relapse therapy suggested that low-dose, long-term, single-agent vinblastine could be as effective as is standard short-term multi-agent chemotherapy in low risk patients. Therefore, proposals are in place to investigate the efficacy of single-agent vinblastine in a new frontline trial for paediatric ALCL. EICNHL proposes to investigate vinblastine as a single-agent frontline treatment in patients negative for minimal disseminated disease (MDD), a prognostic factor previously associated with a lower risk of treatment failure [36,37]. The goal is to assess whether vinblastine could replace the ALCL99 protocol, at least for low risk patients—though it may not improve the OS and EFS rates, the anticipation is that it will be less toxic overall. Patients who can be cured by vinblastine are spared both acute (stomatitis, neutropenia, infections, 1–2% treatment related mortality) and late (risk of secondary malignancies, infertility, cardiac toxicity, obesity, metabolic syndrome) toxicity of the multi-agent chemotherapy which includes etoposide, alkylators, and anthracyclines. A further advantage for single agent vinblastine therapy is that patients can be treated as outpatients. Unfortunately, the long duration of the treatment protocol with weekly hospital visits for 2 years may prove to be a logistical barrier. In addition, this could provide a low toxicity chemotherapy backbone forming a new basis to study the addition of targeted therapies.

### 2.2. Development of Targeted Agents for Frontline Therapy

#### 2.2.1. ALK Inhibition

With EFS and OS rates having barely changed since the NHL-BFM first tested its B-cell NHL protocol on ALCL patients in the 1980s, there is a clear need for new, less toxic therapies for patients in all risk groups (Table 3).

ALK is an ideal drug target particularly as endogenous expression of ALK is limited to neuronal cells during neonatal development [49] which should limit side-effects. However, initial interest in the development of ALK inhibitors was largely non-existent amongst pharmaceutical companies due to not only the favourable survival rates of these patients, but also its orphan disease status. Over a decade after the description of ALK in ALCL, it was identified to be fused to EML4 in 6.7% of Non-Small Cell Lung Cancer (NSCLC) patients as a result of a chromosomal inversion [50] and subsequently the development of ALK inhibitors began. The first phase I clinical trial of Pfizer’s ALK/MET/ROS1 inhibitor, crizotinib, was initiated in 2008 [51]. Other ALK inhibitors have followed, and since crizotinib’s FDA approval in 2011 for advanced ALK-positive NSCLC, ceritinib (Novartis) and alectinib (Hoffmann-La Roche) were likewise approved in 2014 and 2015, respectively [52,53,54]. Two more ALK inhibitors—lorlatinib (Pfizer) and brigatinib (Takeda)—have recently been granted breakthrough therapy designation and FDA-accelerated approval respectively [55]. As usual, there is a necessary lag in the application of novel agents to a paediatric population but these drugs have been slowly filtering through to the treatment of ALCL and other ALK-related malignancies in children.

In Europe, the EICNHL is planning to trial an ALK inhibitor in combination with the ALCL99 backbone as frontline treatment in a phase I safety study. Unfortunately, so far, no ALK inhibitor has been selected or agreed for use in this study [56], although crizotinib is the obvious candidate due to its longer history of use in adults, proven safety, and efficacy in ALK-positive NSCLC. Indeed, crizotinib and combination chemotherapy have already been tested in a phase I trial in children with ALK-related malignancies (NCT01606878), and a trial for adults with ALK-positive ALCL is underway (NCT02419287), and final toxicity data will soon be available from the phase II trial of crizotinib administered in combination with multi-agent chemotherapy in the USA (NCT01979536, Table 3).

The other potential candidate is ceritinib, although its use is associated with significant toxicities, which may limit its application in a paediatric population; the drug has shown severe gastrointestinal toxicity in 14% of NSCLC patients with diarrhoea, nausea, vomiting, or abdominal pain occurring in 95% of 925 NSCLC patients [57]. However, ceritinib has shown long-lasting responses in three ALK-positive adult ALCL patients who relapsed after anthracycline-based chemotherapy and were included in the expansion cohort of the phase I ASCEND-1 trial (NCT01283516). At the time of the report, patients were still on ceritinib treatment with durations ranging from 20 to 26 months [58]. Likewise, a phase I dose escalation trial of single-agent ceritinib in paediatric patients with ALK-expressing malignancies (NCT01742286), showed two out of two ALK-positive ALCL patients to achieve a complete response [59]. Currently, the results of a rare indications, phase II, open-label, multi-centre, multi-arm study (ASCEND-10, NCT02465528) which recruits patients starting from 1 year of age diagnosed with an ALK-positive malignancy other than NSCLC, are expected in 2019 [60]. Interestingly, a phase I/II open-label dose-finding study of ceritinib combined with brentuximab vedotin (BV; discussed later in this review) for frontline treatment of ALK-positive ALCL patients 12 years and older, is planned to open to recruitment in 2018 (NCT02729961). This trial will provide important information regarding new targeted agent combination strategies not involving standard chemotherapy.

Other ALK inhibitors are at earlier stages of development for the treatment of adults with ALK-positive malignancies other than NSCLC and therefore are likely to be slower in percolating through to the treatment of paediatric populations. For example, alectinib, lorlatinib, brigatinib and entrectinib (Ignyta) have shown promising results in ALK-positive NSCLC but have not yet been sufficiently tested in the paediatric setting and long-term toxicities are unknown. The advantage of these ALK inhibitors over crizotinib and ceritinib is that they are able to cross the blood–brain barrier and as such are active against CNS disease [61,62,63,64,65]. However, unlike advanced ALK-positive NSCLC, which is characterized by a high risk of CNS metastases and a high frequency of brain metastases at diagnosis [66], paediatric ALCL patients have a low risk of CNS progression [67].

#### 2.2.2. Targeting CD30

The consistent expression of CD30 (a protein expressed almost exclusively on activated B and T cells) in ALCL provides another therapeutic target [68,69]. One of the earliest attempts was the development of an anti-CD30 monoclonal antibody called BerH2 and a single-chain variable fragment (scFv) linked to immunotoxins saporin-6 and pseudomonas exotoxin A, respectively [70,71]. A few other such therapies have since been trialled in various types of HL with some success [72], but the high reported toxicities make this unsuitable for ALCL. A similar approach that utilized an iodine-131 labelled murine anti-CD30 monoclonal antibody in HL achieved partial remissions [73], however toxicity was again found to be a barrier.

The first mouse–human chimeric anti-CD30 antibody, SGN-30, was developed by Seattle Genetics and tested in a phase I/II pilot study in combination with ifosfamide, carboplatin, and etoposide (ICE) in five children with recurrent ALCL (COG-ANHL06P1, NCT00354107). However, serious adverse events (pleural effusion, ascites, decrease in neutrophil count, capillary leak syndrome, skin and subcutaneous tissue disorders) led to the termination of the study [40].

The activity of SGN-30 was further improved by conjugation with the anti-microtubule agent monomethylauristatin E (MMAE). The resulting antibody–drug conjugate brentuximab vedotin (BV, SGN-35) binds to CD30 on the cell surface initiating its internalization, followed by trafficking to the lysosomal compartment with eventual release of MMAE via proteolytic cleavage [74]. Binding of MMAE to tubulin disrupts the microtubule network, induces cell cycle arrest and results in apoptotic death of the CD30-expressing cell [75]. An initial phase I clinical trial of BV (NCT00430846) was conducted in adults with CD30-positive lymphomas that had failed systemic chemotherapy. The two adult patients with ALCL enrolled into the study both achieved complete remission (CR) [76]. Following this, a phase II study of BV in adults with relapsed or refractory systemic ALK-positive and ALK-negative ALCL was initiated (NCT00866047) [77], and in 2011 BV was approved by the FDA for the treatment of relapsed ALCL following failure of at least one multi-agent chemotherapy protocol for adults. An update to this pivotal study provided 4-year follow-up of patients included in the phase II study; the median Progression-Free Survival (PFS) was 20 months (25.5 months for ALK-positive ALCL patients) and the 4-year OS was 64% [78].

Both crizotinib and BV have since been studied in adults with HL (NCT02243436, NCT01578967, NCT02098512, NCT01874054, NCT00848926 NCT02298283, NCT02227433, NCT02939014, NCT01716806) [79,80,81,82] and NHL (NCT01805037, NCT02462538, NCT01657331, NCT01909934, NCT01352520, NCT01950364, NCT02139592, NCT02419287, NCT02939014, NCT00866047, NCT02280785) in the frontline setting with promising results. Additionally, BV and combination chemotherapy has been trialled in young patients with newly diagnosed HL (NCT02166463).

This has encouraged a randomized phase II COG study for paediatric ALCL, (COG-ANHL12P1, NCT01979536) that compares the use of BV to the ALK inhibitor crizotinib administered with a common chemotherapy backbone (Table 3). This study is the first frontline trial of these targeted agents specifically for children with ALCL. The trial enrolled its first patient in 2013 and final results will be available by the end of 2020; to date, 110 patients have been enrolled and updated study results were presented at the EICNHL meeting in November 2017. The BV arm has been closed, as recruitment is now complete. The crizotinib arm has re-opened at the time of the writing of this manuscript following an FDA-imposed clinical hold in March 2017 due to the occurrence of thrombosis. Catheter-related clots and pulmonary emboli occurred in 10 patients, after which the study committee initially closed the crizotinib arm. This is surprising as the robust and sustained activity observed in the Phase I/II COG-ADVL0912 trial (discussed below) provided the rationale for combining crizotinib at 165 mg/m^2^ with conventional chemotherapy. The only grade 3 or 4 drug-related adverse event was a decrease in neutrophil count occurring in 83% of patients treated at 165 mg/m^2^ crizotinib [83]. In the future, single-agent vinblastine may provide a lower toxicity chemotherapy backbone as mentioned above.

## 3. Treatment of Refractory/Relapsed Disease

### 3.1. Stem Cell Transplantation

Consolidation of chemotherapy response in relapsed and refractory ALCL with allogeneic Stem Cell Transplantation (SCT) remains a subject without international consensus. Some retrospective studies suggest that OS is over 50% for ALK-positive ALCL relapse cases when treated with SCT or continued multi-agent chemotherapy [34,84,85], the former being the standard of care for children or adolescents with some other forms of relapsed or refractory NHL (except ALCL) [84,85]. Therefore, one treatment option for relapsed or refractory ALCL is SCT and four retrospective studies have been conducted to assess its efficacy.

The NHL-BFM working group was the first to report that SCT is a viable option for relapsed ALCL looking back at ALCL patients treated in the 1990s [85,86]. Two retrospective Japanese studies also found that relapsed or refractory ALCL patients who received SCT did better than those who did not [84,87]. For all cases, the risk profile was acceptably low, but this approach was reserved for high-risk ALCL patients who had already relapsed at least once. One of the Japanese studies in particular showed that 30% of relapsed patients treated with chemotherapy alone relapsed a second time, which is similar to the 37.5% of patients treated with autologous SCT who relapsed a second time [84]. However, the patient group was fairly small with only 10 and eight patients treated in each arm, respectively. Allogeneic SCT was more successful, with all six patients entering remission [84]. Collectively, these limited data suggest that allogeneic SCT is superior to autologous SCT [87]. A retrospective French trial also showed mixed results, with 45% of patients treated with autologous SCT entering remission, as opposed to 52% treated with chemotherapy alone [33]. However, there is no consensus on the type of conditioning that should be used for allogeneic SCT with varying regimens of radiotherapy being the more commonly used options.

Two clinical trials are ongoing to test the efficacy of SCT against other second- or third-line treatment options. The planned Japanese trial, JPSLG-ALCL-RIC-18, will specifically test the efficacy of Reduced Intensity Conditioning (RIC) to prepare for allogeneic SCT. EICNHL-ALCL-RELAPSE (NCT00317408), which, between 2004 and 2011 enrolled 80 relapsed paediatric ALCL patients sorted into three arms depending on CD3 expression and time to relapse, tested allogeneic SCT and autologous SCT with and without BEAM (carmustine, etoposide, cytarabine and melphalan)-conditioning in comparison to single agent, weekly vinblastine. EICNHL-ALCL-RELAPSE has no published results. In conclusion, none of the therapies trialled to date have provided decisive data as to how to treat relapsed disease, although the results of the Japanese trial are awaited to inform on the importance of different conditioning regimens.

### 3.2. Development of Future Treatments for Relapsed/Refractory ALCL

#### 3.2.1. ALK Inhibition

As mentioned above, COG was the first group to open a phase I dose-escalation study of an ALK inhibitor (COG-ADVL0912, NCT00939770) [42]. In this trial, crizotinib was administered orally, twice daily in 28-day cycles as a single agent for an indefinite duration, to paediatric patients with ALK-positive relapsed or refractory ALCL that had received at least one course of chemotherapy [43]. Those with relapsed ALCL achieved an objective response rate of 90% [83] when treated with the recommended phase II dose (RP2D) of 280 mg/m^2^ [42]. The 10 patients treated at the RP2D in phase I of the study were included in the phase II study. The additional 10 patients that were treated at the RP2D were specifically enrolled in the phase II expansion cohort. Of the 20 patients included in the phase II expansion cohort, 13 responded within 4 weeks of initiating treatment and the remaining seven within 5 to 8 weeks with CR in 18 out of 20 patients. Two patients came off therapy after experiencing adverse events (AEs), three after experiencing disease progression, 12 proceeded to SCT and two continued on crizotinib [83].

Three years later, a phase I study was also initiated by COG (COG-ADVL1212, NCT01606878) combining crizotinib with conventional chemotherapy for relapsed or refractory paediatric ALCL patients, which provided the requisite safety and tolerability data for eventually integrating crizotinib into frontline treatment regimens for children with ALCL. Preliminary study results are expected in 2018.

In Japan, trials UMIN000016991 and UMIN000028075 are investigating the efficacy and safety of alectinib or crizotinib, respectively, as monotherapies for children with recurrent or refractory ALK-positive ALCL [44,45,46]. UMIN000016991 is the first trial to test an ALK inhibitor other than crizotinib in paediatric ALCL patients. Results for UMIN000016991 and UMIN000028075 are expected in 2020 and 2022, respectively.

**Figure 1:**
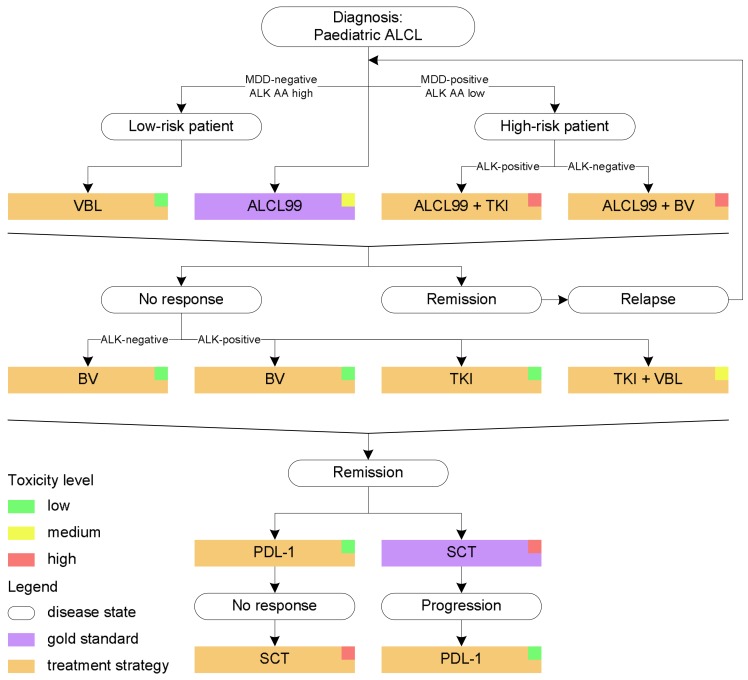
Management of childhood Anaplastic Large Cell Lymphoma (ALCL). ALK AA = ALK auto-antibody; BV = brentuximab vedotin; MDD = minimal disseminated disease; SCT = stem cell transplantation; TKI = tyrosine kinase inhibitor; VBL = vinblastine.

In Europe, an Innovative Therapies for Children with Cancer (ITCC) trial is in progress to treat relapsed patients with ALK-, ROS1- or MET-positive malignancies (not limited to ALCL) with crizotinib either as a single agent or in combination with vinblastine (only patients with ALCL) in a phase IB safety study. The trial (ITCC053, CRISP) will determine the RP2D of vinblastine in combination with crizotinib by dose escalation of vinblastine with a fixed dose of 150 mg/m^2^ crizotinib. Patients will receive a maximum of 24 cycles corresponding to two years of therapy. Salvage of non-responding patients is anticipated by transfer of patients to the EICNHL-ALCL-Nivo trial discussed below (Figure 1).

Even though the final results from the ALK inhibitor trials are still to come, single-agent crizotinib has not yet proven curative, since abrupt relapses following crizotinib discontinuation have been described in isolated cases [88], and no successful reported case of continuous CR after discontinuation of treatment has been reported thus far. Hence, accumulating evidence suggests that ALK inhibitors might have to be taken life-long and drug resistance is a distinct possibility that may require the cycling of patients through different ALK inhibitors as is the case for NSCLC [89,90]. However, crizotinib is currently used to induce second remission in adult relapsed/refractory ALKpositive ALCL patients before allogeneic or autologous SCT [91] (Figure 1).

#### 3.2.2. Targeting CD30

As mentioned earlier, following encouraging results from adult ALCL trials (NCT00430846, NCT00866047), a company-sponsored international phase I/II study of BV in paediatric patients with relapsed or refractory systemic ALCL (NCT01492088) was opened in 2012. Participants received BV at 1.4 mg/kg in Phase I and 1.8 mg/kg in Phase II, on day 1 of every 21-day cycle for up to 16 cycles. Of the 17 ALCL patients recruited into the phase II expansion cohort of the trial, the Overall Response Rate was 53% and time to progression was 6.3 months. However, 13 patients did not complete the study; one patient died and 12 patients dropped out for unspecified reasons. The most common reported drug-related AE was a decrease in neutrophil and lymphocyte counts with one patient experiencing pyrexia and four of 17 patients developing neutralizing anti-therapeutic antibodies [41]. Additionally, a major clinical consideration is cumulative peripheral neuropathy that was observed in 36% of patients recruited into the dose finding study of BV for adults with CD30-positive hematologic malignancies (NCT00430846) [92]. Given the neurologic side-effects of BV, prolonged treatment may be difficult to manage in paediatric patients. Thus, this drug is currently mostly used as a bridge to transplant in relapsed patients (Figure 1).

Interestingly, experimental treatment using imatinib, which inhibits enzymatic activity of PDGFRA, PDGFRB, and c-KIT, resulted in the full remission of a refractory late-stage ALK-positive ALCL patient [93]. This is yet another promising compound that is already being investigated in combination with BV in adult ALK-positive ALCL patients (NCT02462538).

Another recent CD30 targeting therapy uses T-cells engineered to express CD30 Chimeric Antigen Receptors (CAR-T cells). CAR-T cells have already been granted breakthrough designation by the FDA for relapsed or refractory myeloma, and three clinical trials are currently ongoing in CD30-positive relapsed lymphomas in adults (NCT02259556, NCT01316146, NCT02274584). Preliminary results for eight [94] and 18 [95] patients have shown CAR-T cells to be reasonably safe and remissions of variable lengths were achieved. However, the authors note a large number of adverse events and the safety profile has yet to be tested in paediatric patients [96].

#### 3.2.3. Immunotherapy

Accumulating evidence indicates that the immune system plays a major role in the pathogenesis of ALK-positive ALCL [97,98]. Indeed, it has been shown that ALK-positive ALCL cell lines strongly express the cell surface protein, Programmed Cell-Death Ligand 1 (PD-L1; CD274, B7-H1), as determined at both the mRNA and protein levels [99]. Furthermore, immunostaining of ALK-positive ALCL primary patient tumours showed strong PD-L1 expression [100]. Analysis revealed that PD-L1 expression is induced by the chimeric NPM-ALK tyrosine kinase, via STAT3, confirming a unique function for NPM-ALK as a promoter of immune evasion by inducing PD-L1 [100]. PD-1 and its ligands, PDL-1 and PDL-2, have been shown to be involved in immune suppression with increased expression of PD-1 leading to decreased activation of reactive T-cells inhibiting the PI3K/Akt pathway on ligation by ligand [101,102,103].

These observations provided a strong rationale to use consolidative anti-PD1/PD-L1 immunotherapy for relapsed or refractory ALK-positive ALCL. Indeed, three case reports describe a dramatic and durable response using the anti-PD1 monoclonal antibodies pembrolizumab (Merck, Merck Kenilworth, NJ, USA) or nivolumab (Bristol-Myers Squibb, New York, NY, USA) for ALCL patients [104,105,106]. The first, an adult with ALK-negative ALCL was treated with pembrolizumab following chemotherapy, BV and SCT [104]. The second, a 19-year-old ALK-positive ALCL patient was treated with nivolumab following chemotherapy, BV, crizotinib, and SCT [105]. Finally, a case report observed a similar dramatic response to nivolumab in a relapsed 17-year-old patient with ALK-positive ALCL after two lines of treatment including chemotherapy and crizotinib [106]. While the 19-year old developed grade 2 pneumonitis, there are no reports of AEs for the other two patients, pointing towards an acceptable toxicity profile. Only the 17-year-old patient was tested for PD-1 expression on tumour cells by immunostaining showing strong expression throughout the tumour. It should be noted that several publications have shown that PD-1 inhibitors can provoke a response even in tumours which do not have strong PD-1 expression, but also that they sometimes fail in tumours which do show strong PD-1 expression [107]. The lack of an obvious biomarker for PD-1 inhibitor efficacy may make clinical decisions difficult when assessing therapeutic approaches for relapsed disease.

With a clear need for a randomized trial of anti-PD-1 monoclonal antibodies in refractory or relapsed ALCL, ALCL-Nivo has been designed as a phase II trial of nivolumab in paediatric and adult relapsed or refractory ALK-positive ALCL patients. The trial will test the objective response to nivolumab at 24 weeks, for patients who have already relapsed on chemotherapy and either an ALK inhibitor or BV. Should there be sufficient response in this first cohort, the trial also plans to test nivolumab as a consolidation therapy after CR of at least two months as a replacement to SCT. Patients in both cohorts are to be treated with 24 months of Nivolumab at 3 mg/kg every two weeks, and every four weeks after the first eight weeks for patients in the second cohort [48].

Another immunotherapy under investigation, potentially of therapeutic use for ALCL at all stages, is the application of cancer vaccines. Strong expression of the ALK chimera in the majority of ALCL cases combined with near-absent expression of ALK in healthy tissues makes it an ideal candidate for vaccine development. Autoantibodies against ALK as well as cytotoxic and helper T cell responses to ALK have been detected in patients with ALK-positive ALCL both at diagnosis and during remission with a significant inverse correlation between ALK-autoantibody titres and the incidence of relapse [108,109]. Vaccination using a truncated cDNA of ALK has been reported to induce potent and long-lasting protection from local and systemic lymphoma growth in mice [110,111], but this has yet to be trialled in ALCL patients.

## 4. Conclusions

ALCL is susceptible to multiple targeted agents, which highlights the potential to transform the therapy and outcomes for this disease. However, ALCL is a rare lymphoma and the increasing number of possible agents presents a challenge for investigators to select the most appropriate ones. This review has highlighted the agents at the forefront of current investigations in paediatric ALCL. It is likely that, due to the increasing number of malignancies in which aberrant ALK signalling is implicated, there will be yet more targeted and other drugs developed that could be relevant to paediatric ALCL. This should provide the possibility for continued refinement of therapy to achieve the highest survival rates with the least toxicity.

## Figures and Tables

**Table 1:** Treatment outcomes for paediatric patients with Anaplastic Large Cell Lymphoma (ALCL) after frontline multi-agent chemotherapy with or without methotrexate (MTX) or vinblastine (VBL). IDM-HiDAC = intermediate dose MTX-high-dose cytarabine, Chemo. = multi-agent chemotherapy.

Therapy	Study Designation	Paediatric Patients	Treatment Duration (Months)	EFS (Year)	OS (Year)	Grade 3/4 Toxicity	Ref.
Chemo.	NHL-BFM83, 86	62	2–5	81% (9)	83% (9)	N/A	[19]
	HM89	82	8	66% (3)	83% (3)	N/A	[13]
	UKCCSG-B-NHL-9001, -9002/9602, -9003	72	N/A	59% (5)	65% (5)	One toxic death	[18]
	POG9315 (APO arm)	85	11	71% (5)	88% (4)	neutropenia/thrombocytopenia (35%)	[16]
	POG9315 (IDM-HiDAC arm)	90	11	71% (4)	88% (4)	neutropenia/thrombocytopenia (70%)	[16]
	CCG-5941	86	11	68% (5)	80% (5)	neutropenia (82%), thrombocytopenia (66%), anaemia (38%)	[17]
	LNH-92	55	11	69% (5)	74% (5)	neutropenia, hepatic events	[20]
	NHL-BFM90 (K1 arm)	9	2–3	100% (5)	N/A	N/A	[15]
	NHL-BFM90 (K2 arm)	65	2–3	73% (5)	N/A	N/A	[15]
	NHL-BFM90 (K3 arm)	14	4–5	76% (5)	N/A	N/A	[15]
	EICNHL-ALCL99 (MTX1-arm)	175	4–5	74% (2)	90% (2)	hematologic toxicity (79%), infection (50%), stomatitis (21%)	[10]
	EICNHL-ALCL99 (MTX3-arm)	177	4–5	75% (2)	95% (2)	hematologic toxicity (64%), infection (32%), stomatitis (6%)	[10]
Chemo. + VBL	HM91	82	7	66% (3)	83% (3)	N/A	[13]
	EICNHL-ALCL99-VBL	110	17–18	70% (2)	94% (2)	neutropenia (29%)	[21]
	ANHL0131 (APO arm)	64	12	74% (3)	84% (3)	neutropenia (39%), infections (22%)	[22]
	ANHL0131 (APV arm)	61	12	79% (3)	86% (3)	neutropenia (84%), infections (43%)	[22]

**Table 2:** Treatment strategies for childhood ALCL. ARA-C = cytarabine; BV = brentuximab vedotin; Cyc = cyclophosphamide; CZ = crizotinib; Daun = daunorobicin; Doxo = doxorubicin; Eto = etoposide; IDM-HiDAC = intermediate dose MTX high-dose Cytarabine; Ifo = ifosfamide; I/T = intrathecal; IV = Intravenous; MTX = methotrexate; TT = topotecan; VBL = vinblastine; VCR = vincristine; VND = Vindesine. Not detailed in the table: prednisone, prednisolone, dexamethasone and food supplements. * Randomized into MTX1 or MTX3 arm. Shaded area indicates drugs used in the protocol.

Trial Acronym	Other	Cyc	Ifo	Doxo	Eto	MTX (I/T)	MTX (IV)	ARA-C (IV)	ARA-C (I/T)	VCR	VND	VBL	Ref.
HM89													[13]
HM91													[13]
NHL-BFM90 (K1/2 arm)													[15]
NHL-BFM90 (K3 arm)													[15]
POG9315 (APO arm)													[16]
POG9315 (IDM-HiDAC arm)													[16]
CCG-5941													[17]
LNH-92	+Daun												[20]
NHL-BFM95 (R1/2)													[24]
NHL-BFM95 (R3/4)													[24]
EICNHL-ALCL99 (MTX1-arm)													[10]
EICNHL-ALCL99 (MTX3-arm)													[10]
EICNHL-ALCL99-VBL						*							[21]
ANHL0131 (APO arm)													[22]
ANHL0131 (APV arm)													[22]
COG-ADVL1212 (Course A/C/D)	+CZ +TT												[25]
COG-ADVL1212 (Course B)	+CZ												[25]
COG-ANHL12P1 (Course A)	+CZ/BV												[26]
COG-ANHL12P1 (Course B)	+CZ/BV												[26]

**Table 3:** Past, ongoing, and planned clinical trials for paediatric ALCL. Allo = allogeneic; AC = alectinib; auto = autologous; BEAM = carbustine, etoposide, cytarabine and melphalan; BV = brentuximab vedotin; CR = ceritinib; CZ = crizotinib; Cyc = cyclophosphamide; ARA-C = cytarabine; Dexa = Dexamethasone; Doxo = doxorubicin; Eto = etoposide; Ifo = ifosfamide; MTX = methotrexate; SCT = stem cell transplantation; TT = topotecan; VBL = vinblastine; VCR = vincristine. * as stated on the ClinicalTrials.gov webpage.

Stage	ClinicalTrials.gov Identifier	Trial Acronym	Treatment	Phase	Time Frame*	Location	No *	Ref.
Front-line	NCT00006455	EICNHL-ALCL99	ALCL99 (Cyc, MTX, Ifo, Eto, ARA-C, Doxo) +/− VBL	III	1999–2005	Europe, Japan	487	[21,31]
	NCT00059839	COG-ANHL0131	APO (Doxo, MTX, VCR) +/− VBL	III	2003–2014	USA	125	[22]
	NCT01979536	COG-ANHL12P1	CZ/BV + (Dexa, Ifo, MTX, ARA-C, Eto)/(Dexa, MTX, Cyc, Doxo)	II	2013–2020	USA	140	[26]
	NCT02729961	NCI-2016-00396	BV+CR	I/II	2017–2023	USA	30	[38]
Relapse	NCT00317408	EICNHL-ALCL-RELAPSE	Allo SCT/BEAM-conditioning + auto SCT/VBL	N/A	2004–2014	Europe	96	[39]
	NCT00354107	COG-ANHL06P1	SGN-30, Ifo, Carboplatin, Eto	I/II	2007–2010	USA	5	[40]
	NCT01492088	C25002	BV	I/II	2012–2018	Worldwide	36	[41]
	NCT00939770	COG-ADVL0912	CZ	I	2009–2020	USA	26	[42,43]
	NCT01606878	COG-ADVL1212	CZ + (Cyc, TT)/(VCR, Dexa, Doxo)	I	2013–2018	USA	65	[25]
	N/A	UMIN000016991	AC	II	2015–2020	Japan	10	[44,45]
	N/A	UMIN000028075	CZ	I/II	2017–2022	Japan	23	[46]
	N/A	ITCC053/CRISP	CZ +/− VBL	IB	2016–2021	Europe	82	[47]
	N/A	EICNHL-ALCL-Nivo	Nivolumab	II	Planned	Europe	38	[48]
